# Role of Uric Acid Metabolism-Related Inflammation in the Pathogenesis of Metabolic Syndrome Components Such as Atherosclerosis and Nonalcoholic Steatohepatitis

**DOI:** 10.1155/2016/8603164

**Published:** 2016-12-14

**Authors:** Akifumi Kushiyama, Yusuke Nakatsu, Yasuka Matsunaga, Takeshi Yamamotoya, Keiichi Mori, Koji Ueda, Yuki Inoue, Hideyuki Sakoda, Midori Fujishiro, Hiraku Ono, Tomoichiro Asano

**Affiliations:** ^1^Division of Diabetes and Metabolism, Institute for Adult Disease, Asahi Life Foundation, 1-6-1 Marunouchi, Chiyoda-ku, Tokyo, Japan; ^2^Department of Medical Science, Graduate School of Medicine, Hiroshima University, 1-2-3 Kasumi, Minami-ku, Hiroshima City, Hiroshima, Japan; ^3^Division of Neurology, Respirology, Endocrinology and Metabolism, Department of Internal Medicine, Faculty of Medicine, University of Miyazaki, Miyazaki 889-1692, Japan; ^4^Department of Internal Medicine, Graduate School of Medicine, University of Tokyo, 7-3-1 Hongo, Bunkyo-ku, Tokyo, Japan; ^5^Department of Endocrinology and Diabetes, School of Medicine, Saitama Medical University, Moroyama, Saitama 350-0495, Japan

## Abstract

Uric acid (UA) is the end product of purine metabolism and can reportedly act as an antioxidant. However, recently, numerous clinical and basic research approaches have revealed close associations of hyperuricemia with several disorders, particularly those comprising the metabolic syndrome. In this review, we first outline the two molecular mechanisms underlying inflammation occurrence in relation to UA metabolism; one is inflammasome activation by UA crystallization and the other involves superoxide free radicals generated by xanthine oxidase (XO). Importantly, recent studies have demonstrated the therapeutic or preventive effects of XO inhibitors against atherosclerosis and nonalcoholic steatohepatitis, which were not previously considered to be related, at least not directly, to hyperuricemia. Such beneficial effects of XO inhibitors have been reported for other organs including the kidneys and the heart. Thus, a major portion of this review focuses on the relationships between UA metabolism and the development of atherosclerosis, nonalcoholic steatohepatitis, and related disorders. Although further studies are necessary, XO inhibitors are a potentially novel strategy for reducing the risk of many forms of organ failure characteristic of the metabolic syndrome.

## 1. Introduction

**Figure 1:**
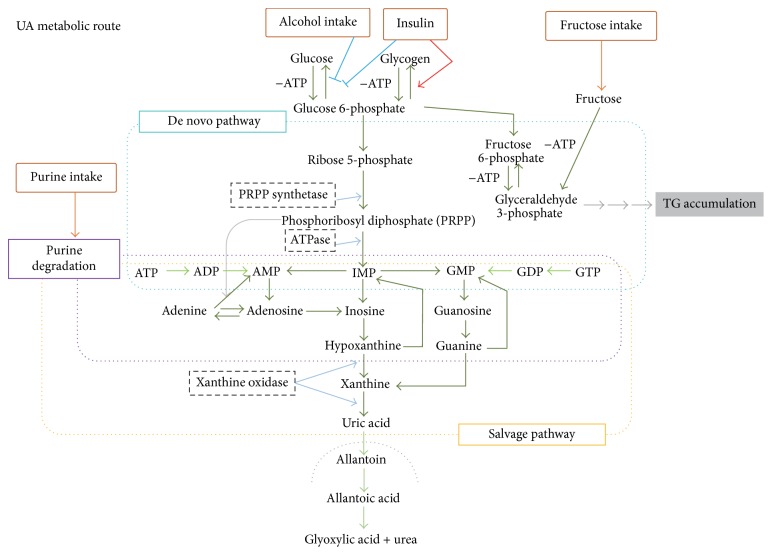
Metabolic pathways involving UA.

Uric acid (UA) is the end product of the metabolic pathway for purines, the main constituents of nucleotides. The pathway of UA generation is shown in Figure 1. Briefly, inosine monophosphate (IMP) is derived from de novo purine synthesis and from purine salvage. Hypoxanthine from IMP is catalyzed to xanthine and then to uric acid by xanthine oxidase (XO). De novo nucleotide synthesis generates IMP via ribose-5-phosphate, catalyzed to 5-phosphoribosyl-1-pyrophosphate (PRPP). In the salvage pathway, hypoxanthine-guanine phosphoribosyl transferase (HGPRT) plays an important role in generating IMP, thereby inhibiting UA generation.

Since humans are unable to catabolize UA to the more soluble compound allantoin due to lack of urate oxidase or uricase [1], the serum UA concentration is higher in humans than almost all other mammals. However, this high UA level in humans has been regarded as being beneficial in the presence of elevated oxidative stress [2]. UA is oxidized to allantoin and other metabolites via nonenzymatic oxidation [3] and, thus, UA can function to neutralize prooxidant molecules, such as hydroxyl radicals, hydrogen peroxide, and peroxynitrite. UA shows the highest scavenging rate constant against O_2_^−•^, with constants being low against CH3^•^ and t-BuOO^•^ [4]. UA directly (nonenzymatically) and preferentially deletes nitric oxide (NO) and forms 6-aminouracil in physiological environments or in association with antioxidants [5]. In vitro, UA has both an antioxidant effect on native LDL and a prooxidant effect on mildly oxidized LDL [6]. Allantoin does not have these effects. The mechanisms of these reactions vary among combinations of prooxidant molecules and solution polarities [7].

It has been suggested that this antioxidant effect of the high UA concentrations in humans contributes to neuroprotection in several neurodegenerative and neuroinflammatory diseases [8–14].

However, despite the potential antioxidant effect of UA itself, numerous studies have revealed close associations of serum UA concentrations and various disorders, most of which are included in the metabolic syndrome category. Thus, UA metabolism may be a so-called double-edged sword as regards the inflammatory and/or oxidative responses in many organs, though on the whole, its harmful effects appear to outweigh the benefits of UA in most cases.

In this review, we first explain the two putative molecular mechanisms underlying inflammation occurrence in relation to UA metabolism; one is inflammasome activation via UA crystallization and the other involves superoxide free radicals generated by XO. While the UA crystallization mechanism would be dependent on a high serum UA concentration, the latter may not necessarily reflect the serum UA concentration though XO activity does lead to the production of reactive oxygen species (ROS).

Subsequently, lines of research showing relationships between UA metabolism and the development of various disorders are introduced and discussed. Importantly, recent studies have demonstrated beneficial effects of XO inhibitors against the occurrence and/or progression of several disorders, particularly atherosclerosis and nonalcoholic steatohepatitis (NASH), both of which are associated with insulin resistance, hyperlipidemia, and/or obesity. In this review, atherosclerosis and NASH are discussed extensively, while studies of gout and chronic kidney diseases (CKD) are mentioned briefly. In conclusion, we propose that such XO inhibitors may be more useful for preventing a variety of disorders, such as atherosclerosis and NASH, than previously believed, probably via an anti-inflammatory effect.

## 2. Inflammation Occurrence Related to UA Metabolism

Among the disorders related to hyperuricemia, gout is the most representative and well known. Features of gout include painful arthritis affecting the limbs, caused by reduced UA crystals in the joints. While symptoms of a gout attack are typical of an acute inflammatory response, as indicated by the presence of swelling, heat, rubescence, and pain, there are many disorders with mild but chronic inflammation which are very likely to be related to UA metabolism. In the latter case, superoxide free radicals generated by XO are key players leading to chronic inflammatory processes eventually resulting in impaired organ functions. Thus, we introduce two independent mechanisms underlying UA metabolism-induced inflammation.

### 2.1. Inflammasome Activation by Crystallized UA Particles

In 2002, the inflammasome concept was proposed to involve multiple proteins and to control the cleavage of prointerleukin 1 (IL-1) [15]. Initially, inflammasomes were considered to play a role in immune responses and serve as defense systems against pathogens [16, 17]. However, a line of subsequent studies has elucidated that inflammasomes are key players in the onsets of a wide range of diseases as well as host defense. Excessive metabolites, such as ATP or monosodium urate crystals (MUC), were also confirmed to be involved in the activation of inflammasomes, and inflammatory responses occurring via inflammasomes have been demonstrated to be linked to the onset and progression of human diseases, including gout, atherosclerosis and NASH, as described below in detail [18–24].

Inflammasomes are known to be divided into discernible patterns, depending on component proteins [16]. Among them, the NLRP3 inflammasome, comprised of three major components, Nod-like receptor 3 (NLRP3), apoptosis-associated speck-like protein containing a CARD (ASC) and caspase-1, has been well investigated. Maturations of both IL-1 and IL-18 by inflammasomes require a two-step mechanism. First, the Toll-like receptor ligands, such as lipopolysaccharide (LPS), activate the NF-*κ*B pathway and upregulate the expression levels of interleukins, including pro-IL-1*β* and pro-IL-18. Subsequently, the inflammasome complex activated by pathogen-associated molecular patterns (PAMPs) or damage-associated molecular patterns (DAMPs) cleaves pro-IL-1*β* or pro-IL-18, resulting in the production of mature interleukins [15–17].

**Figure 2:**
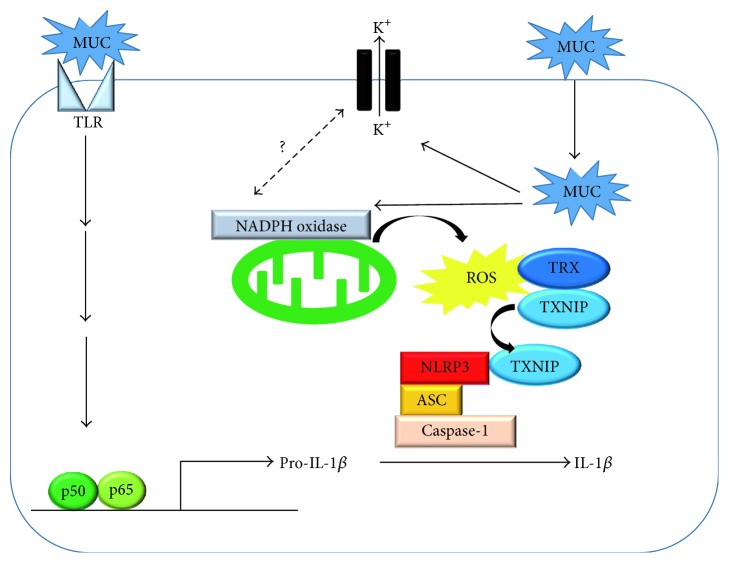
MUC induces inflammasome activation. MUC activates the NF-*κ*B pathway through TLR2/4, thereby increasing the expressions of pro-IL-1*β* or pro-IL-18. At the same time, MUC induces ROS release from mitochondria. The generated ROS detaches TXNIP from thioredoxin and enables TXNIP to interact with the NLRP3 complex. The binding of TXNIP to NLRP3 activates inflammasomes, leading to the production of mature IL-1*β* or IL-18. MUC: monosodium urate crystals, TLR: Toll-like receptor, TXNIP: thioredoxin-interacting protein, TXR: thioredoxin, and ROS: reactive oxygen species.

MUC also reportedly serve as a danger signal and trigger the activation of inflammasomes [18]. Although the mechanism of inflammasome activation by MUC has not been fully elucidated, the following mechanism was proposed. MUC stimulate the Toll-like receptor 2/4-Myd88 pathway and raise transcriptional levels of pro-IL-1*β* through the NF-*κ*B pathway [25]. It is theorized that MUC-induced inflammasome activation is driven by two key factors. One is a decrease in the intracellular potassium concentration. Indeed, the addition of high potassium abrogated IL-1*β* release by MUC. The other is the generation of ROS, because an antioxidant, N-acetyl-cysteine, abolished IL-1*β* secretion by MUC [26]. Other studies have indicated the application of MUC to raise intracellular ROS levels. However, the relationship between intracellular K^+^ level changes and ROS generation remains unknown, and future studies are expected to resolve this issue [27, 28]. Elevation of intracellular ROS mediates the detachment of thioredoxin-interacting protein (TXNIP) from thioredoxin and enables TXNIP to associate with NLRP3, leading to NLRP3 inflammasome activation [29, 30]. Thus, MUC accumulation promotes inflammatory responses through inflammasomes (Figure 2) and thereby promotes the onset of diseases, such as gout.

### 2.2. Superoxide Free Radicals Generated by XO

When mammalian xanthine dehydrogenase (XDH) is converted to XO under stressed conditions such as tissue damage and ischemia [31], superoxide anion and hydrogen peroxide are produced during molybdenum hydroxylase-catalyzed reactions in a molar ratio of about 1 : 3 [32]. The proteolytic activation from XDH to XO is required for superoxide generation [33]. In essence, XO oxidizes a variety of purines and pterins, classified as molybdenum iron-sulfur flavin hydroxylases. When XO reacts with xanthine, electrons are transferred from Mo, Fe-S, and FAD. XO produces FADH2, while XDH produces FADH. Only FADH2 reacts with O2 [34]. In the UA metabolic pathway, XO oxidizes hypoxanthine from nucleic acid metabolites into xanthine and xanthine into UA (Figure 1). XO, as well as nicotinamide adenine dinucleotide phosphate (NADPH) oxidase and the mitochondrial electron-transport chain, generates ROS [35].

ROS from XO might play physiological roles, especially in development. Treatment during pregnancy with allopurinol alters maternal vascular function involving *β*1-adrenergic stimulation and impairs the fetal *α*1-adrenergic vasoreflex response involving NO [36]. Fetal XO is activated in vivo during hypoxia and XO-derived ROS contributes to fetal peripheral vasoconstriction, leading to fetal defense against hypoxia [37]. XO depletion induces renal interstitial fibrosis, and renal epithelial cells from XOR (−/−) mice are more readily transformed into myofibroblasts [38]. Indeed, how ROS from XO directly and physiologically acts in vivo is unknown.

The tissue and cellular distributions of XO in mammals are highest in the liver and intestines due to XO-rich parenchymal cells [39]. Xanthine oxidoreductase (XOR) is present in hepatocytes, while XO is present in bile duct epithelial cells, concentrated toward the luminal surface. Moreover, in human liver disease, proliferating bile ducts are also strongly positive for XO [40]. Molybdenum supplementation significantly increased XO activities in the liver and small intestinal mucosa [41]. XO activity is low in human serum, the brain, heart, and skeletal muscle, while being rich in microvascular endothelial cells [42] and is also present in macrophages [43]. Circulating XO can adhere to endothelial cells by associating with endothelial glycosaminoglycans [44]. The study using electron spin resonance measurements revealed the contribution of increased XO activity to endothelial dysfunction in patients with coronary artery diseases [45].

XO activation is induced by LPS, angiotensin II, NADPH oxidase, hypoxia, hypoxia-inducible factor 1, and inflammatory cytokines such as IL-1*β* [46–49]. The release of XO is increased in hypercholesterolemia, chronic hyperammonemia, thermal trauma, beta-thalassemia, brain ischemia, and pulmonary artery hypertension [50–54]. Aging is another factor associated with elevated XO activity. Indeed, XO was significantly higher in the aortic walls and skeletal muscles of old rats than in those of their young counterparts. The correlation between plasma XO activity and age is observed in both humans and rats [55]. It appears that hyperglycemia itself has no impact on liver XO activity, though cardiac, renal, and brain XO activities were shown to be increased in rats with advanced diabetes [56, 57]. XO activity rises remarkably in ischemic congestive heart failure and XO localizes within CD68 positive macrophages [43]. The association between XO and ischemic reperfusion injury has been well investigated. XO is one of the major superoxide sources in ischemia/reperfusion injuries of the heart [58], forebrain [59], skin [60], liver [61, 62], and gastric mucosa [63], as well as multiple system organ failure after hind limb reperfusion [64]. XO activity, along with lipid peroxidation, myeloperoxidase activity and NO levels, is increased in the liver in response to renal ischemia/reperfusion in diabetic rats [65]. Ischemia/reperfusion injury is attributable to elevated XO activity and ATP depletion related to increasing hypoxanthine and xanthine levels during ischemia, and reperfusion provides O_2_ for oxidation of these compounds [1].

Superoxide production by XO may also be enhanced by increasing the amount of its substrate, purine bodies. Excess fructose metabolism results in ATP depletion which is associated with degradation of AMP to hypoxanthine, followed by conversion to UA by XO [66]. Indeed, the serum UA level is upregulated in response to a fructose burden [67]. Inversely, UA stimulates fructokinase and fructose metabolism during fatty liver development [68]. ATP depletion, such as that characteristic of glycogen storage disease type 1 [69], hypoglycemia [70], exercise [71], and starvation [72], also increases UA production. Conditions associated with DNA turnover, such as tumor progression [73] and tumor lysis [74], are also mediated by XO.

Superoxide produced by XO is an important messenger inducing inflammation and signal transduction, leading to tissue damage. We found inflammatory cytokines to be induced via XO when foam cells form with lipid accumulation [75]. XO regulates cyclooxygenase-2 [76] in the inflammatory system, and XO appears to be critical for innate immune function [77]. XO increased Egr-1 mRNA and protein, as well as the phosphorylation of ERK1/2, while pretreatment with an ERK1/2 inhibitor prevented induction of Egr-1 by XO [78]. In addition, XO reportedly reduced SUMOylation of PPAR*γ* in inflammatory cells [79]. ROS from XO augment TRB3 expression in podocytes [80].

**Figure 3:**
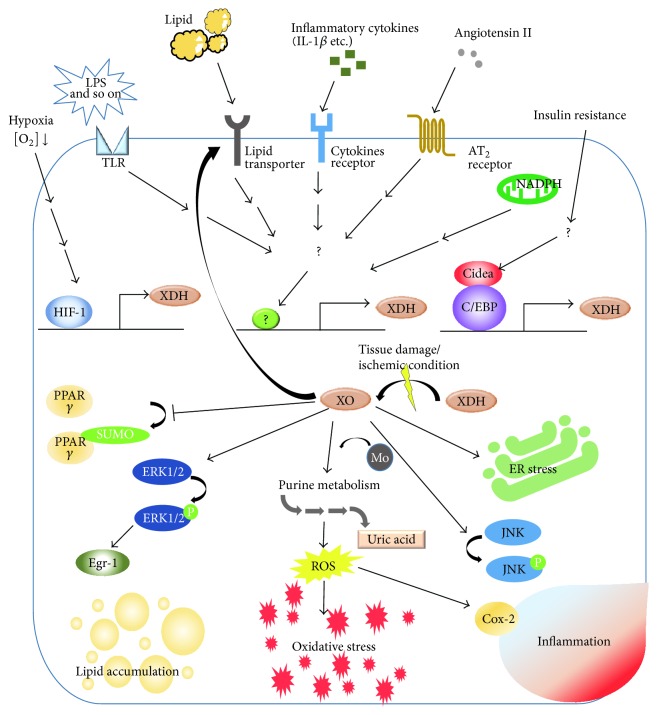
Involvement of XO in molecular pathologies related to inflammation; “causes and results.”

As noted above, superoxide from XO has been suggested to play roles in various forms of inflammatory or ischemic pathophysiology (Figure 3), not necessarily involving hyperuricemia.

## 3. UA Metabolism and Chronic Renal Disease, Atherosclerosis, Heart Failure, and NASH

While gout is a disorder well known to be caused by the precipitation of UA crystals, the involvement of hyperuricemia in CKD is also widely recognized. The major causes of CKD have been regarded as diabetes mellitus and hypertension, and thus, hyperuricemia was long viewed as a consequence of CKD. In fact, loss of kidney function reduces the excretion of UA into urine, resulting in hyperuricemia. In contrast, recent studies demonstrated a significant association between serum UA and the development of CKD. While each metabolic syndrome component, including hyperglycemia, hyperlipidemia, and hypertension, was associated with an increased CKD risk, hyperuricemia was apparently an independent risk factor not influenced by the others. Therefore, hyperuricemia is both a cause and a consequence of CKD and is frequently associated with other metabolic syndrome features.

In terms of CKD pathogenesis, serum UA is likely to activate the renin-angiotensin system resulting in vascular smooth muscle cell proliferation [81] and to induce an epithelial-to-mesenchymal transition of renal tubular cells [82]. XO inhibitor treatment reportedly reduced intercellular adhesion molecule-1 (ICAM-1) expression in tubular epithelial cells [83] of mice. We speculate that UA itself and superoxide free radical generation both play roles in the molecular mechanisms underlying hyperuricemia-related CKD development, but further research is required to elucidate the complex mechanistic interactions between serum UA and CKD.

As mentioned in Section 2, both UA and superoxide free radicals are simultaneously produced by XO and might be the pathophysiological cause of these diseases. As shown in Figure 3, chronic inflammation is also involved in pathophysiological processes, generally exhibiting a close relationship with oxidative stress. ROS from XO induces LPS-induced JNK activation via inactivation of MAPK phosphatase- (MKP-) 1 [84] and XO regulates cyclooxygenase-2, one of the master regulators of inflammation [76]. Therefore, damage from UA, ROS, and UA-induced and/or ROS-induced inflammation might together contribute to the progression of certain diseases, and distinguishing which mechanism acts first is often difficult in lifestyle-related diseases.

### 3.1. Atherosclerosis, Vascular Dysfunction, and Heart Failure

Although the relationships between serum UA levels and atherosclerotic diseases, including hypertension [85, 86], have been documented, whether or not serum UA itself is an independent cardiovascular risk factor remains controversial as most hyperuricemic patients with cardiovascular diseases (CVD) have other complications such as hypertension, dyslipidemia, diabetes, and CKD as well, which are generally regarded as more established risk factors for CVD than hyperuricemia. Recently, however, a growing body of evidence from both clinical and basic research supports the hypothesis that hyperuricemia, partly via elevated XO activity, is an independent risk factor for hypertension and CVD.

Despite the association between hyperuricemia and hypertension having been recognized since the 19th century [85], it was not until recently that hyperuricemia was demonstrated to be an independent risk factor for hypertension development [87–93]. A recently published meta-analysis showed that the adjusted relative risk of developing hypertension was 1.48 for hyperuricemic patients [94], and this association was apparently much stronger in younger, early-onset hypertensive patients [86, 95]. Several clinical trials have demonstrated the beneficial effects of UA lowering therapy for hypertension [96–99]. In a trial targeting prehypertensive obese adolescents, administration of either allopurinol (XO inhibitor) or probenecid (uricosuric agent) lowered blood pressure [98]. Consistently, both allopurinol and benziodarone (uricosuric agent) reduced blood pressure in rats with hypertension induced by hyperuricemia [100, 101], suggesting that not only XO activity but also UA itself plays an important role in the pathogenesis of hypertension.

Besides the association with hypertension, hyperuricemia or gout has been confirmed to be related to the morbidity and the mortality of CVD [102–106]. According to a recently published meta-analysis [107], the relative risks of morbidity and mortality for coronary heart diseases were 1.13 and 1.27, respectively, in hyperuricemic patients as compared to controls. Several clinical studies have indicated the benefits of XO inhibitors for reducing the incidence of myocardial infarction [108], improving exercise tolerance in patients with stable angina [109], and enhancing endothelial function [110, 111]. However, interestingly, unlike the case of treating hypertension, uricosuric agents have failed to show any benefits in patients with hyperuricemia or gout [110, 112].

What are the mechanisms underlying the aforementioned association between hyperuricemia and atherosclerotic diseases? First, the role of XO in the pathogenesis of atherosclerosis merits attention. As described above, XO produces ROS when converting hypoxanthine into xanthine and then UA. XO is also expressed in endothelial cells [113] and was shown to be increased in the aortic endothelial cells of ApoE^−/−^ mice [114], an established model of atherosclerosis. Since oxidative stress inactivates NO and leads to endothelial dysfunction [115], endothelial XO, especially given its enhanced expression during the development of atherosclerosis, contributes to vascular damage via ROS production.

Recently, we established that XO activity in macrophages also plays a key role in the development of atherosclerosis [75]. During atherosclerosis development, monocytes migrate beneath the endothelium and transform into macrophages, which then turn into foam cells by incorporating modified low density lipoproteins (LDL) (such as oxidized LDL and acetyl LDL) or very low density lipoproteins (VLDL). Foam cells contribute to the formation of unstable plaques by secreting inflammatory mediators and matrix-degrading proteases (such as matrix metalloproteinases (MMPs)) and by generating a prothrombotic necrotic core by eventually undergoing necrotic or apoptotic death [116]. We demonstrated that allopurinol treatment ameliorated aortic lipid accumulation and calcification of the vessels of ApoE-KO mice and that allopurinol markedly suppressed the transformation of J774.1 murine macrophages or primary cultured human macrophages into foam cells in response to stimulation with acetyl LDL or VLDL. The expressions of scavenger receptors (SR-A1, SR-B1, and SR-B2) and VLDL receptors in J774.1 cells were upregulated by XOR overexpression and downregulated by siRNA-mediated XOR suppression, raising the possibility that XO activity in macrophages positively regulates foam cell formation by increasing the uptake of modified LDL or VLDL. Conversely, expressions of ABCA1 and ABCG1, which regulate cellular cholesterol efflux, were decreased by XOR overexpression and increased by XOR knockdown. Furthermore, allopurinol suppressed the expressions of inflammatory cytokines such as IL-1*β*, IL-6, IL-12, and TNF*α*, and the expressions of VCAM1, MCP-1, and MMP2, which were upregulated in J774.1 cells transformed into foam cells by atherosclerogenic serum. Subsequently, febuxostat, another XO inhibitor, was also demonstrated to attenuate the development of atherosclerotic lesions in ApoE^−/−^ mice [114]. That study showed XO expression to be increased in macrophages infiltrating atherosclerotic plaques and that febuxostat diminished the ROS level in the aortic walls of ApoE^−/−^ mice. The authors demonstrated that cholesterol crystals (CCs) increased endogenous XO activity and ROS production in macrophages and that CCs enhanced not only IL-1*β* release via NLRP3 inflammasome activation but also secretions of other inflammatory cytokines such as IL-1*α*, IL-6, and MCP-1 from macrophages, processes which in turn were suppressed by febuxostat or ROS inhibitors. The significance of NLRP3 inflammasome activation in macrophages by CCs was verified by the observation that atherosclerosis in high-cholesterol diet fed LDL receptor- (LDLR-) deficient mice was alleviated by transplanting bone marrow from NLRP3-deficient, ASC-deficient, or IL-1*α*/*β*-deficient mice [117]. Taking these observations together, we can reasonably speculate that XO in macrophages enhances foam cell formation, ROS production, and NLRP3 inflammasome activation, all three of which exacerbate inflammation and plaque formation, thereby contributing to the development of atherosclerotic diseases [75, 114–116].

Independently of XO, UA itself is widely recognized to exert direct effects on vascular functions. Vascular endothelial cells express several UA transporters [118] and incorporated UA impairs NO production and leads to endothelial dysfunction [118, 119]. In vascular smooth muscle cells, UA stimulates proliferation and ROS production and inhibits NO production via increased angiotensin II expression [81, 120]. As noted above, not only XO inhibitors but also uricosuric agents markedly lowered blood pressure, especially in studies targeting early-stage hypertensive patients [98] and those using animal models [100, 101]. The results obtained suggest that UA presumably contributes to early-stage hypertension by promoting renal vasoconstriction via reduced NO production and activation of the renin-angiotensin system [86, 98].

### 3.2. Nonalcoholic Steatohepatitis

The number of nonalcoholic fatty liver disease (NAFLD) patients including those with NASH has been increasing worldwide and a portion of NASH patients will progress to hepatocarcinoma onset [121–123]. Therefore, numerous investigations have been performed in efforts to elucidate the causes of NASH.

NASH is characterized by fat deposition, inflammation and fibrosis in the liver, and a two-hit mechanism of onset has been proposed [124–126]. This hypothesis is that fatty liver formation and subsequent injuries, including inflammation and oxidative stress, cause NASH pathology [127]. Interestingly, recent studies have raised the possibility that UA is among the risk factors for NASH pathology. We discuss the relationship between UA and NASH below.

#### 3.2.1. Serum UA Is a Predictor of NAFLD/NASH Onset and Progression

Many clinical studies have been carried out to investigate the relationship between serum UA levels and NAFLD/NASH progression. For example, a cohort study in Korea found the serum UA level to be a useful marker for predicting NAFLD development because the serum UA concentration correlated positively with the 5-year incidence rate of NAFLD [128]. Their conclusion is supported by another study showing that serum UA levels of NAFLD patients are higher than those of control groups [129]. In addition, there are also studies demonstrating that serum UA is a risk factor for the development and/or progression of NAFLD including NASH [130–132].

Consistent with these observations, hepatic XO activities and serum UA levels are reportedly increased in murine NAFLD/NASH models [133, 134]. Moreover, a fraction of NAFLD/NASH patients also have obesity, and hypertrophic adipocytes were also reported to secrete UA [135]. Taken together, these results indicate serum UA to be a good parameter for predicting the development of NAFLD/NASH, and that XO inhibitors or uricosuric agents might have potential as treatments for ameliorating the features of NAFLD.

#### 3.2.2. The Mechanism of UA-Induced NAFLD/NASH Progression

As described above, increasing serum UA or XO activity apparently plays important roles in NAFLD/NASH onset and progression. Interestingly, UA was reported to induce fat depositions by enhancing lipogenesis in hepatocytes. Fructose treatment of HepG2 cells reportedly increased both the intracellular UA concentration and triglyceride (TG) accumulation, while allopurinol, an XO inhibitor, suppressed this fructose-mediated TG deposition. Moreover, the application of UA alone was demonstrated to increase intracellular TG contents as well as ROS generation in mitochondria [136]. As a mechanism of UA-induced TG accumulation, the authors asserted that the elevation of intracellular ROS by UA raised both the citrate concentration and ATP citrate lyase activity via enhanced phosphorylation at S455, resulting in the induction of lipogenesis. These observations are supported by those of another study in which pretreatment with antioxidants inhibited the elevation of triglyceride contents by UA [137]. The authors asserted that ROS generation by UA evoked endoplasmic reticulum stress, leading to upregulation of lipogenic genes, such as acetyl CoA carboxylase1 and FASN [137].

ROS generation by UA is considered to depend on NADPH oxidase activation [136, 138, 139]. For example, UA reportedly promotes translocation of the NADPH oxidase subunit NOX4 into mitochondria [136]. It was also reported that UA treatment raises NADPH oxidase activity and alters its localization, leading to lipid oxidation [139]. In addition, XO may also function as a source of ROS generation because XO activity is upregulated in the livers of murine NASH models.

Collectively, these observations indicate that UA enhances fatty acid synthesis by regulating lipogenesis and induces ROS generation by regulating NADPH oxidase activity and upregulating fatty acid synthesis, thereby contributing to NASH development.

#### 3.2.3. Inflammasome Participation in NASH Progression

As described elsewhere, UA is involved in inflammasome activation. Recent investigations have provided convincing evidence that inflammasomes are key players in NASH development. An initial study revealed that inflammasome impairment exacerbated the NASH progression induced by feeding a methionine-choline deficient diet for 4 weeks to ASC or IL-1 KO mice [140]. Subsequent studies, however, found that inflammasomes themselves exacerbate NASH symptoms. For example, it was reported that NLRP3 deficiency prevents liver fibrosis in response to a choline diet deficient in amino acids [141]. In addition, caspase-1 deficient mice were also resistant to developing steatosis or fibrosis while being fed a high-fat diet [142]. Moreover, other groups have demonstrated that diets which lead to NASH also increase the expressions of inflammasome components [143–145].

Taking these lines of evidence together, in the initial stage of NASH, inflammasomes appear to exert a protective effect, but continuous inflammasome activation appears to cause excessive productions of inflammatory cytokines, ultimately resulting in liver injury. Although, to date, numerous factors playing important roles in NASH progression have been identified, UA also appears to be a key participant in the onset of NAFLD/NASH.

### 3.3. Insulin Resistance, Diabetes, and Hyperlipidemia

Hyperuricemia was reportedly found to be related to insulin resistance in several clinical analyses [146–152]. In addition, several meta-analyses have suggested the UA level to be positively associated with the development of type 2 diabetes mellitus (DM) [153–156], although Mendelian randomization studies did not support circulating UA as being among the causes of DM development [157, 158]. In metabolic syndrome patients, an oxidative stress marker, the myeloperoxidase level, was decreased by allopurinol and endothelial function improved [159]. On the other hand, rapid UA reduction achieved by rasburicase, a urate oxidase, in obese subjects with high UA resulted in increasing the markers of systemic and skeletal muscle oxidative stress while having no effect on insulin sensitivity [160].

Furthermore, excess fructose intake is one of the major causes of the development of obesity with hyperuricemia, fatty liver, and metabolic syndrome. Fructose is metabolized by fructokinase to fructose-1-phosphate and results in a drop in both intracellular phosphate and ATP levels [161]. The intracellular phosphate decrease stimulates AMP deaminase (AMPD), the enzyme catalyzing the degradation of AMP to inosine monophosphate and eventually UA. Activated AMPD increases the expressions of gluconeogenesis genes, that is, PEPCK and G6Pase, via inhibition of AMP-activated protein kinase (AMPK) [162]. AMPD also increases lipogenesis through AMPK inhibition. AMPK phosphorylation was decreased in HepG2 cells treated with UA. The UA increased fructose-induced TG accumulation and decreased *β*-hydroxybutyrate levels, dose-dependently, while allopurinol, a XO inhibitor, blocked it. Because UA is the downstream product of AMPD and allopurinol abolished fructose-induced lipid accumulation, AMPD effects on AMPK appeared to depend on UA [163]. UA activates the transcription factor ChREBP, which triggers a vicious cycle of fructokinase transcription and accelerated fructose metabolism [68]. Via these mechanisms, activated AMPD and increased UA production tend to promote fat accumulation and glucose production.

UA is considered to be an antioxidant in human blood, though UA induces oxidative stress in cells [164]. UA raised NADPH oxidase activity and ROS production in mature adipocytes. The stimulation of NADPH oxidase-dependent ROS by UA resulted in the activation of MAP kinase p38 and ERK1/2, a decrease in NO bioavailability, and increases in both protein nitrosylation and lipid oxidation [138]. Increased UA production, in turn, generates mitochondrial oxidants. Mitochondrial oxidative stress inhibits aconitase in the Krebs cycle, resulting in citrate accumulation and the stimulation of ATP citrate lyase and fatty acid synthase, ultimately leading to de novo lipogenesis [136]. In hepatocytes treated with high UA, oxidative stress is increased, which activates serine (rat Ser307 and human Ser312) phosphorylation of IRS-1. This activity impairs Akt phosphorylation, thereby resulting in acute hepatic insulin resistance after exposure to high UA levels [165]. Therefore, UA-induced lipid accumulation and oxidative stress are responsible for the development of insulin resistance and diabetes.

## 4. Beneficial Effects of XO Inhibitors

**Figure 4:**
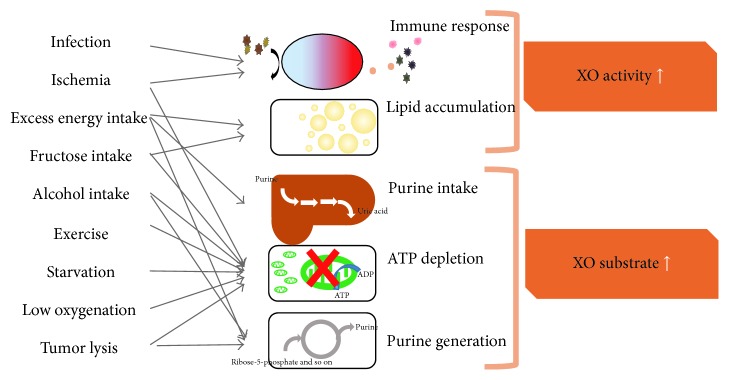
Increased catalyst activity of XO, originating from pathological and physiological events. Involvement of XO in pathophysiological processes suggests applications of XO inhibitors to the treatment of various disorders.

Involvement of increased XO catalyst activity in pathophysiological processes (Figure 4) suggests applications of XO inhibitors to the treatment of various disorders. At present, XO inhibitors, including allopurinol, oxypurinol, febuxostat, and topiroxostat, are widely used for treating gout and hyperuricemia. Furthermore, XO inhibitors have been experimentally or clinically shown to exert beneficial effects by lowering serum UA and oxidative stress.

Febuxostat preserved renal function in 5/6 nephrectomized rats with and without coexisting hyperuricemia and prevented diabetic renal injury in streptozotocin-treated rats [166, 167]. Febuxostat also ameliorated tubular damage, diminished macrophage interstitial infiltration, and suppressed both proinflammatory cytokine activities and oxidative stress [168]. Febuxostat also reduced the induction of endoplasmic reticulum stress, as assessed by GRP-78 (glucose-regulated protein-78), ATF4 (activating transcription factor-4), and CHOP (C/EBP homologous protein-10) [169]. The clinical significance of measuring the serum UA level and XO inhibition for renal protection has largely been established by the results of recent studies [170–173].

On the other hand, beneficial effects of XO inhibitors on atherosclerosis and NASH constitute an evolving concept that has yet to be proven. In rats with fructose-induced metabolic syndrome, febuxostat treatment reversed hyperuricemia, hypertension, dyslipidemia, and insulin resistance [174]. The beneficial effects of XO inhibitors on NASH are rarely reported, except by our research group [134], because animal models of NASH with obesity, inflammation, and fibrosis have been difficult to establish. NASH in response to the MCD diet, as used in our studies, caused primarily inflammation and also made the mice lean, such that no benefit of XO inhibition was obtained [134]. Thus, we next used a high-fat diet containing trans-fatty acids and a high-fructose diet to induce NASH development in our animal models. Another report showed that inhibition of XO activity also significantly prevents hepatic steatosis induced by a high-fat diet in mice. XO has also been indicated to regulate activation of the NLRP3 inflammasome [175].

Atherosclerosis has been far more extensively investigated than NASH, both clinically and experimentally. Tungsten, acting as an XO inhibitor, has an inhibitory effect on both atherosclerosis and oxidative stress [176]. We reported for the first time that more specific XO inhibition, using allopurinol rather than tungsten on macrophages, resulted in the inhibition of foam cell formation and reduced atherosclerotic lesions in ApoE-KO mice, independently of the serum lipid profile [75]. We also identified phenotypic changes of macrophages in response to allopurinol, such as alterations of gene expressions involved in lipid accumulation. Moreover, both XO overexpression and knockdown of XO expression revealed VLDL receptors to be dramatically upregulated by XO. Febuxostat was also proven to have similar effects in terms of reducing the atherosclerotic lesions in ApoE-KO mice, and oxidative stress was reduced in macrophages from atherosclerotic lesions [113]. Febuxostat also suppressed LPS-induced MCP-1 production via MAPK phosphatase-1-mediated inactivation of JNK [84]. As a strategy for suppressing atherosclerosis, XO inhibition is expected to act on either macrophages or inflammatory cells.

XO inhibitors also improve endothelial function and prevent vascular remodeling. Oxypurinol reduces O_2_^−^ radical dot production and improves endothelial function in blood vessels from hyperlipidemic experimental animals [69]. XO inhibition can also provide protection from radiation-induced endothelial dysfunction and cardiovascular complications [177]. Allopurinol treatment prevents hypoxia-induced vascular remodeling in the lung [178]. However, controversy persists as to whether the effect of XO on endothelial function is clinically relevant as an interventional target [49]. Pretreatment with XO inhibitors has beneficial effects on ischemia/reperfusion injuries of the intestine [179], in the impaired liver [61, 62], the edematous brain [180], kidneys with contrast induced nephropathy [181], and coronary ischemia [182]. XO inhibitors prevent postischemic O_2_^−^ generation [183].

## 5. Conclusion

Inflammation related to UA metabolism is induced via either inflammasome activation by UA crystal precipitation or free radical production in response to XO activity. In addition to gout, many disorders are known to be related to UA metabolism and XO inhibitor treatments have been shown to be effective for preventing the onset and/or the progression of such disorders. In particular, atherosclerosis and NASH are diseases for which relationships with UA metabolism were not immediately recognized, but rodent model studies revealed the importance of UA metabolism maintenance for managing these disorders. We believe the impact of UA metabolism on many diseases accompanying chronic inflammation to have been underestimated. Future studies are anticipated to reveal the pathological contribution of serum UA and/or XO activity to the specific processes underlying various disorders. Further study of the detailed molecular mechanisms is clearly warranted.

## Abbreviations

UA: Uric acid
MUC: Monosodium urate crystal
NASH: Nonalcoholic steatohepatitis
XO: Xanthine oxidase
LPS: Lipopolysaccharide
TIMP: Tissue inhibitor of metalloproteinases
MCP-1: Monocyte chemoattractant protein 1
NADPH: Nicotinamide adenine dinucleotide phosphate
CKD: Chronic kidney disease
ICAM-1: Intercellular adhesion molecule-1.
